# Compatible solutes from hyperthermophiles improve the quality of DNA microarrays

**DOI:** 10.1186/1472-6750-7-82

**Published:** 2007-11-23

**Authors:** Nicoletta Mascellani, Xiuping Liu, Simona Rossi, Jlenia Marchesini, Davide Valentini, Diego Arcelli, Cristian Taccioli, Mauro Helmer Citterich, Chang-Gong Liu, Rita Evangelisti, Giandomenico Russo, Jorge M Santos, Carlo M Croce, Stefano Volinia

**Affiliations:** 1 Dipartimento di Morfologia ed Embriologia and DAMA, Data Mining for Analysis of DNA Microarrays, Telethon Facility, Università degli Studi di Ferrara, Ferrara, Italy; 2 Comprehensive Cancer Center, Ohio State University, Columbus, OH 43210, USA; 3 Nucleic Acid Facility, IDI – IRCCS, Roma, Italy; 4 STAB VIDA Lda, Oeiras, Portugal; 5 Department of Medical Epidemiology and Biostatistics, Karolinska Institute, Stockholm, Sweden; 6 ECBio S.A., Oeiras, Portugal

## Abstract

**Background:**

DNA microarrays are among the most widely used technical platforms for DNA and RNA studies, and issues related to microarrays sensitivity and specificity are therefore of general importance in life sciences. Compatible solutes are derived from hyperthermophilic microorganisms and allow such microorganisms to survive in environmental and stressful conditions. Compatible solutes show stabilization effects towards biological macromolecules, including DNA.

**Results:**

We report here that compatible solutes from hyperthermophiles increased the performance of the hybridization buffer for Affymetrix GeneChip^® ^arrays. The experimental setup included independent hybridizations with constant RNA over a wide range of compatible solute concentrations. The dependence of array quality and compatible solute was assessed using specialized statistical tools provided by both the proprietary Affymetrix quality control system and the open source Bioconductor suite.

**Conclusion:**

Low concentration (10 to 25 mM) of hydroxyectoine, potassium mannosylglycerate and potassium diglycerol phosphate in hybridization buffer positively affected hybridization parameters and enhanced microarrays outcome. This finding harbours a strong potential for the improvement of DNA microarray experiments.

## Background

In recent years DNA microarrays as other high throughput molecular techniques became first choice investigation methods for DNA and RNA studies. Early applications included expression profiling and DNA mutation analysis [1]. Recently, single nucleotide polymorphisms (SNPs) and comparative genomics hybridization also found widespread solutions in microarray based assays [2-4]. The optimization of the microarray workflow, including the hybridization step, is thus a primary target for the evolution of more efficient protocols.

The identification of compatible solutes in hyperthermophilic microorganisms, and of their stabilization effect, prompted us to test their effectiveness in microarray protocols. The accumulation of low molecular mass compounds is known to be a common strategy used by microorganisms to survive in environmental and stressful conditions [5]. Hyperthermophiles accumulate compatible solutes (the so-called hypersolutes) rarely encountered in mesophiles. These solutes are generally negatively charged, whereas mesophiles accumulate primarily neutral solutes.

Mannosyl glycerate (MG) is a compatible solute accumulated by some thermophiles and hyperthermophiles in answering to osmotic aggressions. Mannosyl glycerate was initially identified in marine red algae of the family *Ceramiales*, and then in Archaea bacteria [6] and it was shown to be a good enzyme stabilizer [7-10]. Recently, MG was also found to be a very effective nucleic acids stabilizer during frost preservation and transport. MG stabilizing properties are shared by some of its synthetic derivatives like mannosyl lactate (ML). In turn, diglycerol phosphate (DGP) is a new and rare hypersolute from *Archaeoglogus *and it displays remarkable properties of protein stabilization [11,12]. Ectoine (ECT) and its derivative hydroxyectoine (HECT) were found in halophile organisms where they play the role of proteins and nucleic acids protectors, as well as free radicals suppressors [13].

The use of osmolytes to improve protein stability is a well established practice. On the contrary no reports have yet demonstrated the effect of hypersolutes on nucleic acids hybridization *in vitro*. To test the effect of hypersolutes on DNA hybridization, we have chosen the Affymetrix system, currently one of the most used and tested microarray platforms. These chips consist of hundreds of thousands oligonucleotides, or more, *in situ *synthesized by a combination of photolithography and oligonucleotide chemistry [14]. In the expression profiling chips that we used here each mRNA transcript is represented by a probe set, i.e. a group of oligonucleotides of around 25 nucleotides in length. This platform is attractive for our purposes because it extends its relevance beyond the RNA expression field. In fact chips with identical technology are also used for SNP detection and for genome re-sequencing. The core element in the Affymetrix design is the perfect match/mismatch probe strategy: for each probe designed to be perfectly complementary to a target sequence, an identical partner probe, except for a single central base mismatch, is generated. These probe pairs allow quantitation and subtraction of signals caused by non specific cross hybridization. Currently, the Affymetrix procedure requires the use of 1 microgram of un-amplified RNA. This RNA amount might still be too high for those studies, where the available sample is limited. Amplification could be performed, in such cases, but it is an expensive and time consuming step in addition to the standard labeling procedure.

The aim of our work was that of verifying whether hypersolutes can further improve this efficient system. Since this platform is well characterized, we could apply proprietary and open source quality control techniques. The results we describe here show that three hypersolutes, HECT, DGP and MG, proved to be very beneficial for the outcome of Affymetrix microarray experiments.

## Results

A preliminary screening of all hypersolutes: non ionic ectoine (ECT) and hydroxyectoine (HECT), potassium salts of diglycerol phosphate (DGP), mannosyl glycerate (MG) and mannosyl lactate (ML) at three different concentrations in the hybridization solution (50, 150 and 300 mM) was carried out. Hydroxyectoine, DGP and MG reduced background, in particular at the lowest concentration, while no significant difference in absolute signal intensity was detected (data not shown). Only these compounds were hence further investigated, and the concentration range extended towards lower values.

Additional hybridizations with the 3 hypersolutes were performed at 10 and 25 mM and repeated at 50 and 150 mM. This plan was set up to investigate the concentration effect and to determine the most effective working concentration of hypersolutes in the hybridization buffer. Each run was carried out in quadruplicate, in addition to a control test (without hypersolute) for each series. Quality assessment by normalized unscaled standard errors (NUSE), relative log expression (RLE) and pseudo images was performed with Bioconductor package affyPLM. All chips passed the quality control (QC) and were included in the following statistical analysis.

Further QC parameters were assessed by using the Affymetrix proprietary tools. Means of raw Q, background, scaling factor and percent present calls values with their standard deviations and p values (from t-test) are reported in Table 1.

**Table 1:** Hypersolutes improve DNA microarray quality parameters

**Compatible solute**	**HECT**				**DGP**				**MG**			
***Concentration (mM)***	***10***	***25***	***50***	***150***	***10***	***25***	***50***	***150***	***10***	***25***	***50***	***150***
Mean raw Q ± SD	2.1 ± 0.05	1.8 ± 0.1	2.6 ± 0.2	2.6 ± 0.2	2.0 ± 0.1	2.0 ± 0.1	2.6 ± 0.4	3.2 ± 0.4	1.9 ± 0.1	2.0 ± 0.1	2.4 ± 0.1	2.5 ± 0.2
% raw Q s *vs *control	+3.1	-8.3	+0.8	+4.6	-0.5	+1.0	+4.2	+26.5	-5.7	-0.4	-4.0	-0.8
p (t-Test s *vs *controls)	0.15	0.04*	0.45	0.19	0.43	0.38	0.31	0.01*	0.06	0.45	0.22	0.44
Mean bkg ± SD	69.9 ± 0.9	61.0 ± 3.4	84.5 ± 9.2	89.2 ± 8.4	67.2 ± 1.9	68.9 ± 3.8	91.0± 14.1	117. ± 18.7	62.2 ± 4.4	67.6 ± 1.2	81.3 ± 8.1	86.8 ± 6.0
% bkg s *vs *control	+3.0	-10.2	-4.1	+1.3	-1.0	+1.4	+3.3	+32.8	-8.4	-0.4	-7.7	-1.4
p (t-Test s *vs *controls)	0.12	0.01*	0.30	0.43	0.35	0.35	0.37	0.02*	0.04*	0.43	0.16	0.42
Mean SF ± SD	3.9 ± 0.3	4.2 ± 0.3	2.9 ± 0.3	3.2 ± 0.2	4.1 ± 0.1	4.2 ± 0.1	3.4 ± 0.6	2.8 ± 0.4	4.1 ± 0.2	4.0 ± 0.2	2.8 ± 0.1	3.2 ± 0.4
% SF s *vs *control	-8.0	-0.1	-9.6	-0.4	-1.7	-0.7	+7.9	-11.0	-1.5	-4.0	-11.4	-1.0
p (t-Test s *vs *controls)	0.04*	0.49	0.17	0.48	0.25	0.40	0.26	0.14	0.33	0.10	0.09	0.46
Mean %P ± SD	29.8 ± 0.4	30.5 ± 1.1	30.4 ± 0.8	29.6 ± 0.7	30.0 ± 0.8	30.1 ± 1.0	30.5 ± 1.6	30.4 ± 1.1	29.7 ± 1.1	29.7 ± 0.9	31.0 ± 1.0	30.8 ± 0.5
%P s *vs *control	+3.0	+5.3	+1.3	-1.1	+3.6	+3.9	+1.9	+1.3	+2.7	+2.5	+3.6	+2.9
p (t-Test s *vs *controls)	0.08	0.04*	0.30	0.33	0.08	0.08	0.29	0.32	0.16	0.17	0.11	0.12

Affymetrix quality control parameters; raw Q, background (bkg), scaling factor (SF) and percent present calls (%P): mean and standard deviation (SD) among replicates; % gain of solute(s) respect to the controls, with relative p values. HECT: hydroxyectoine; DGP: potassium diglycerol-phosphate; MG: potassium mannosylglycerate. * p-value < 0.05

Raw Q and background values were within the acceptable ranges for all the assays and very similar to each other, with the exception of high concentration DGP (150 mM). Twenty five mM HECT and 10 mM DGP and MG resulted in higher sample quality, reduction of arrays surface auto-fluorescence and of nonspecific binding. Individual scaling factors ranged between 3.6 and 4.4 for the 10 and 25 mM series and between 2.4 and 3.9 for the 50 and 150 mM series, and were all acceptable being within two-fold of each other.

One of the most interesting parameters for investigators is probably the percent present calls, defined as the fraction of "expressed" probe sets relative to the total of the array. The higher number of genes can be measured as present (whilst reducing noise and background), the most useful the results will be. HECT, DGP and MG increased percentage of "present" calls, thus improving array sensitivity. This positive effect was more pronounced at low solute concentrations (10 and 25 mM). In particular, the use of 25 mM HECT as additive to hybridization yielded a 5.3 % gain in present calls.

**Figure 1:**
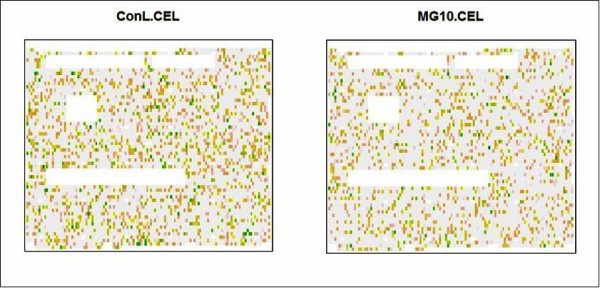
Pseudo-images of the weights for DNA microarrays hybridized in the presence of 10 mM mannosyl glycerate (MG) and the untreated control.

We also used chips pseudo-images in the diagnostics of array. Chips pseudo-images of robust linear model weights are a computational method to measure the quality of microarrays. Areas of poor quality are indicated by green (low weights), while high quality by light grey spots (high weights). An example of the weights' pseudo-images is reported in Figure 1 for chips hybridized in the presence of 10 mM MG (one of the effective hypersolutes and concentrations) and its control. The image plot of the control chip shows a larger number of green areas, corresponding to poor quality, than that of the hypersolute. Thus pseudo-images confirmed that hybridizations in the presence of 10 mM MG produced higher quality microarrays.

**Figure 2:**
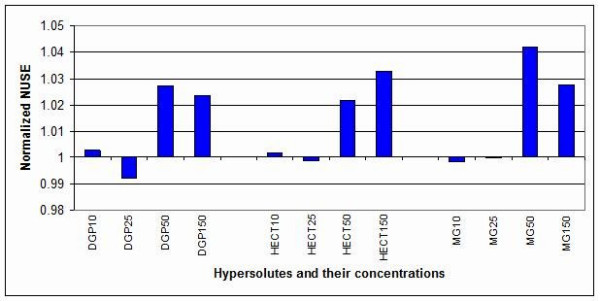
**Normalized unscaled standard errors (NUSE)**. NUSE values were normalized on the controls (1 = 100% = untreated control). DGP25 (25 mM DGP), HECT25 (25 mM HECT) and MG10 (10 mM MG) arrays showed improved NUSE with respect to the control arrays.

**Figure 3:**
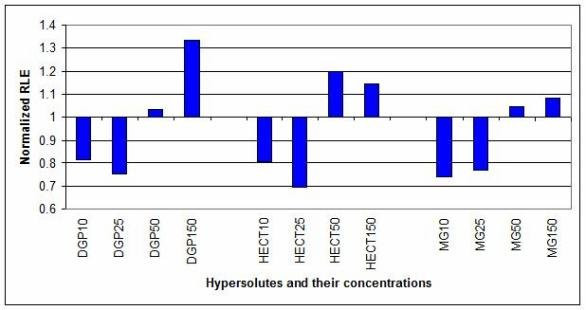
**Relative log expression (RLE)**. RLE values were normalized on the controls (1 = 100% = untreated control). Notice that DGP10 (10 mM DGP), DGP25 (25 mM DGP), HECT10 (10 mM HECT), HECT25 (25 mM HECT), MG10 (10 mM HECT) and MG25 (25 mM MG) arrays displayed improved RLE with respect to the controls.

The graphs of the normalized unscaled standard errors (NUSE) and the relative log expression (RLE) are displayed as bar charts in Figure 2 and 3, respectively. In both cases, the values were normalized on the corresponding controls (the value 1 on the Y axis means 100% of the untreated control), as described in the Methods section. Poor quality chips have normalized NUSEs and RLEs higher than 1 (control value), while high quality chips have normalized NUSEs and RLEs lower than 1. NUSEs and RLEs for almost all 10 and 25 mM compatible solute concentrations were lower than 1, indicating improved arrays quality. Only in two cases, 10 mM DGP and HECT, the NUSEs were slightly higher than controls. On the other hand, the error indexes for the 50 and 150 mM solute concentrations were higher than the controls. The values reported in Figure 2 and 3 were referred to experiments run at the same site.

**Figure 4:**
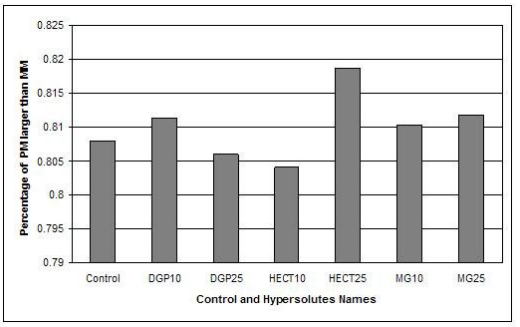
**Percentage of PM > MM**. Mean percentage of PM > MM for each hypersolute (as defined in the Bioconductor package). DGP10 (10 mM DGP), HECT25 (25 mM HECT), MG10 (10 mM MG) and MG25 (25 mM MG) showed higher percentage than control (i.e. 0.80 = 80% of PMs larger than MMs).

The hybridizations were performed on Affymetrix GeneChip Test3 arrays. These chips are commonly used for the assessment of target quality and contain probes representing a subset of genes from different organisms. The fragmented cRNA used in our assays hybridized to the human and the highly conserved probes. The results reported above were obtained by analyzing the whole array. In order to exclude solutes-induced cross-hybridization, we also measured PMs and MMs only for human probes. The mean values of PM > MM confirmed the higher quality of hybridizations with10 mM DGP, 25 mM HECT and 10 and 25 mM MG (Figure 4). Notice that i.e. 0.80 means 80% of PM larger than MM, according to the affy Bioconductor package [15].

## Discussion

The positive effect of compatible solutes in the hybridization of microarrays could be due to the involvement of solutes in different processes. For example, low concentrations of hypersolutes might improve the specificity of DNA:cRNA interactions by destabilizing imperfect double helices, containing unpaired nucleotides. Hypersolutes might on the contrary stabilize the perfect matched pairings typical of the short Affymetrix probes (25 bp or less). The combination of these two effects would thus lead to the higher signal to noise ratio observed in our DGP, MG and HECT hybridizations. Additionally, the reduction in background could be a result of the lower solid support auto-fluorescence. Finally, a stabilization of the cRNA in solution, during the overnight hybridization, might be the last component in the chain of events leading to the improved S/N ratio. The effect of hypersolutes on hybridization efficiency was not related to potassium ions (DGP, MG and ML were all potassium salts) since ML did not show a significant improvement over control (data from the preliminary screening, not reported).

The positive effects of hypersolutes on Test3 hybridizations were displayed both for the total probe sets and for the human specific probe sets. Cross-hybridization induced by compatible solutes was not detectable by our analysis. The small improvement in the percentage of present calls, applied at genome level, would add as many as 500–1000 genes to an expression profile.

Finally, the beneficial role of compatible solutes might be very valuable for other Affymetrix systems, like the SNPs platform. Considering the higher constrains of the SNP chips, genotyping might benefit from compatible solutes even more than expression profiling. It is likely, but remains to be experimentally verified, that different high throughput techniques, either solid state, or beads-based solution systems, might also gain from using compatible solutes.

## Conclusion

Low millimolar concentrations of hydroxyectoine, potassium diglycerol phosphate and potassium mannosylglycerate reduced DNA microarray background and improved hybridization efficiency. The results were highly significant when analyzed by comparing different quality control measures: raw Q, background (bkg), scaling factor (SF), percent present calls (%P), chips pseudo-images, normalized unscaled standard errors (NUSE) and relative log expression (RLE). Twenty five mM DGP, 10 mM HECT and 10 mM MG were shown to be the optimal solutes and concentrations. The experiments were carried out and confirmed in two different Affymetrix facilities. The application of this finding to hybridization protocols could result in a significant improvement of microarray experiments, not limited to expression profiling.

## Methods

Different series of transcriptome analysis using constant human RNAs and variable concentrations of hypersolutes were performed. Total RNA from HEK 293 cells was extracted by using NucleoSpin^® ^RNA II Kit (Macherey-Nagel, Düren, Germany). Different batches of RNA were pooled together after quality assessment by spectrophotometric analysis supported by gel electrophoresis and Agilent Bioanalyzer™ (Palo Alto, CA, USA). The RNA Integrity Numbers (RINs) from the Bioanalyzer™ reports were all between 9.5 and 10.0. Compatible solutes were from BITOP (Witten, Germany).

All operations were carried out according to the standard Affymetrix protocol [16], with the sole exception of adding compatible solutes to the hybridization buffer. The fragmented cRNA targets were hybridized onto Affymetrix GeneChip^® ^Test3 Arrays (Santa Clara, CA, USA). The samples for hybridization were prepared by adding the hypersolute to the fragmented cRNAs in DEPC water. PolyA spike-ins were not used. Arrays were scanned by using the Affymetrix GeneChip^® ^3000 scanner. The CEL files were analyzed using the Affymetrix GeneChip^® ^Operating Software, and standard array quality parameters such as raw Q, background, scaling factor and percent present calls (all defined in the Affymetrix GeneChip^® ^Expression Analysis Technical Manual [17]), were measured. T-test was used to compare means for independent samples.

In addition to the standard Affymetrix quality parameters listed above, we needed additional statistical measures to test chips quality and to evaluate and validate the results. Therefore, we used the Bioconductor package affyPLM [18]. This package performs quality Affymetrix array tests by a variety of procedures, such as pseudo images, standard error evaluation and relative log expression. Chip pseudo-images are very useful for detecting potential quality problems. For each hybridization we produced a pseudo-image, where areas of low quality were green and those of high quality were light grey. Another quality parameter we used was the normalized unscaled standard errors (NUSE). The estimated standard error obtained for each gene on each array from fitPLM was standardized across arrays so that the median standard error for that gene was 1. NUSE statistics (NUSE median and inter-quartile range IQR) were computed for each array. The relative log expression (RLE) was also studied. The RLE values were calculated for each probe-set by comparing the expression value on each array against the median expression value for that probe-set across all arrays. The RLE statistics (RLE median and IQR) were computed for each array.

After computing NUSE and RLE statistics for each array, the results were resumed by M = (median+2*IQR); median represents a measure of central location of the data and IQR (inter-quantile range) is defined as the difference between the 75^th ^percentile and the 25^th ^percentile (i.e. the upper and the lower quantiles). M was used to identify confidence limits to evaluate RLEs and NUSEs. The mean of M measures was calculated for each group of replicates, and finally M means were normalized by the control mean. PM (perfect match) and MM (mismatch) were calculated by using PM and MM affy Bioconductor package functions [15]. The PM/MM based quality comparisons were performed by calculating the percentage of PM larger than MM in each array.

## Authors' contributions

NM helped draft the manuscript and with JM prepared the RNA. XL and CGL carried out the microarray hybridization in the 1st facility directed by CMC. MHC carried out the microarray hybridization in the 2nd facility directed by GR. SR, NM, DV, DA, and CT contributed to data analysis. JMS, NM, and SV conceived the study, participated in its design and coordination, and helped draft the manuscript. All authors read and approved the final manuscript. RE, GR performed critical reading of the manuscript.
